# Effect of NTN and Lmx1*α* on the Notch Signaling Pathway during the Differentiation of Human Bone Marrow Mesenchymal Stem Cells into Dopaminergic Neuron-Like Cells

**DOI:** 10.1155/2021/6676709

**Published:** 2021-07-31

**Authors:** Jinhua Zhang, Bo Yang, Lilin Luo, Linhui Li, Xuantao Yang, Juanjuan Zhang, Yuxin Xie, Wanpu Wang, Shuaiyao Lu

**Affiliations:** ^1^Department of Pathology, The First People's Hospital of Yunnan Province, The Affiliated Hospital of Kunming University of Science and Technology, Kunming, China; ^2^Institute of Medical Biology, Chinese Academy of Medical Sciences and Peking Union Medical College, Kunming, China

## Abstract

Human bone marrow mesenchymal stem cells (h-BMSCs) have the potential to differentiate into dopaminergic neuron-like cells to treat Parkinson's disease. The Notch signaling pathway has been implicated in the regulation of cell fate decisions such as differentiation of BMSCs. This study investigated changes in the expression of Notch-related genes in the differentiation of BMSCs in vitro into dopaminergic (DA) neuron-like cells. BMSCs transfected with empty lentiviral vectors served as the control group and those transfected with NTN and Lmx1*α* recombinant lentiviral vectors served as the experimental group. After induction and culture of NTN and Lmx1*α*-transfected h-BMSCs for 21 days, the cells exhibited features of dopaminergic neuron-like cells, which were observed by transmission and scanning electron microscopy and verified by immunofluorescence of tyrosine hydroxylase (TH) and dopamine transporter (DAT). These induced cells could secrete dopamine and had basic action potentials. Expression of the neural stem cell (NSC) markers, including octamer-binding protein (*Oct4*), paired box gene 6 (*Pax6*), and sex determining region Y-box 1 (*SOX1*), increased on day 14 of induction and decreased on day 21 of induction during differentiation. The human Notch signaling pathway PCR array showed a differential expression of Notch-related genes during the differentiation of h-BMSCs into DA neuron-like cells in vitro relative to that in the control group. In conclusion, h-BMSCs overexpressing NTN and Lmx1*α* can successfully be induced to differentiate into dopaminergic neuron-like cells with a neuronal phenotype exhibiting fundamental biological functions in vitro, and NTN and Lmx1*α* may affect the expression of Notch-related genes during differentiation.

## 1. Introduction

Parkinson's disease (PD) is an age-related neurodegenerative disease that is clinically characterized by the death of dopaminergic (DA) neurons and the formation of Lewy bodies [1, 2]. The mainstay of PD management is drug therapy, but drug therapy cannot prevent disease development. Fortunately, many studies have proved that bone marrow mesenchymal stem cells (BMSCs) are suitable for cell transplantation therapy in PD [3]. In our previous study, we demonstrated that DA can be efficiently induced from BMSCs overexpressing the LIM homeobox transcription factor 1*α* (Lmx1*α*) and neurturin (NTN) [4]. When these BMSCs were grafted into 1-methyl-4-phenyl-1, 2, 3, 6-tetrahydro pyridine- (MPTP-) lesioned right side substantia nigra of rhesus monkeys with PD, they were found to improve impaired behavior [4]. Similarly, Lmx1*α* has been shown to regulate midbrain DA neuronal differentiation, and direct injection of NTN into the midbrain restores degenerating DA neurons [5–9]. However, the exact mechanism by which BMSCs differentiate into DA neurons remains unclear.

Until recently, many reports studying the mechanism of differentiation of BMSCs have focused on signaling pathways such as the Notch, Wnt, and Sox signaling pathways. Notch family components play a considerable role in the regulation of cellular processes ranging from embryonic and postnatal development to adult stem cell differentiation [10–12]. Notch signal transduction mediates adjacent cell-cell interactions. When the Notch receptor combined ligands, the intracellular Notch domain NICD is released from the membrane to activate the expression of transcription factors [13]. No further intermediate steps involving a second messenger and protein kinase are needed. Therefore, focusing on the Notch signaling pathway is the best way to study the mechanism of differentiation of BMSCs [14]. In the present study, we successfully established an h-BMSC cell line overexpressing Lmx1*α* and NTN and attempted to investigate the role of Notch-related genes in the differentiation of BMSCs.

## 2. Materials and Methods

### 2.1. Cell Culture

Human BMSCs (h-BMSCs; HUXMA-01001 cell line) were provided by Cyagen Biosciences (Guangzhou, China) and cultured in growth medium for human BMSCs (Cyagen Biosciences, Guangzhou, China) in air containing 5% CO_2_ at 37°C. The cells were cultured to 90% confluence, digested with trypsin, and divided into two flasks.

### 2.2. Gene Transfection of h-BMSCs

Recombinant Lmx1*α* and NTN plasmids were constructed using lentivirus particles and established by Shanghai Taitool Bioscience Co., Ltd., and the recombinant plasmid containing both these genes was designated PLV-duaI-Lmx1*α*-NTN. h-BMSCs were seeded into the wells of six-well plates at a concentration of 1 × 10^5^ per well, after which the cells were infected with the recombinant and control lentiviruses. The multiplicity of infection (MOI) was set at 10, 50, and 100, and the duration was set at 48 hours to explore maximal transfection efficiency.

### 2.3. Induction of DA from h-BMSCs

Human BMSCs were seeded into six-well plates at a density of 1 × 10^5^ per well in the growth medium. Twenty-four hours later, cultured h-BMSCs were transfected with Lmx1*α* and NTN recombinant lentivirus and cultured in maintenance medium (low-glucose DMEM/F12 with 5% fetal bovine serum and 1% antibiotics). After one day, the medium was changed to the DMEM/F12 medium with 20% fetal bovine serum, 6 mm *β*-mercaptoethanol, and 20 ng/mL *β*-fibroblast growth factor. At 24 h after incubation, in the preinduction medium, h-BMSCs were induced in the induction medium (DMEM/F12 medium containing 15% fetal bovine serum, 6 mm *β*-mercaptoethanol, 20 ng/mL *β*-fibroblast growth factor, 1.7 m recombinant human Sonic hedgehog factor, 100 ng/mL fibroblast growth factor 8, and 0.3 g/mL retinoic acid). The medium was replaced with fresh induction medium every 4-5 days. Fourteen days later, the medium was changed to the differentiation maintenance medium (DMEM/F12 with 2% B27 and 1% N_2_).

### 2.4. Western Blotting

h-BMSCs were transfected with Lmx1*α* and NTN recombinant lentivirus for 48 h and then incubated with 300 *µ*L of RIPA buffer containing 1% protease inhibitor for 10 min at 4°C. The resultant lysates were centrifuged at 12000 rpm for 10 min, and the supernatant was collected and used to determine the protein concentration by BCA assay, according to the instructions of the BCA protein assay kit. Then, 80 *µ*g samples of total protein were separated by electrophoresis in electrophoresis buffer (3.03 g Tris, 18.77 g glycine, and 0.1% SDS) at 60 V for 25 min and 90 V for 3 h and then transferred to polyvinylidene difluoride (PVDF) membranes at 200 Ma for 1 h. The membranes were blocked in 5% bovine serum albumin for 1 h and incubated with primary antibodies against Lmx1*α* (1 : 1000, ab139726, Abcam), NTN (1 : 500, ab8061, Abcam), and glyceraldehyde-3-phosphate dehydrogenase (GAPDH, 1 : 1000, P30008, Abmart) in Tris-buffered saline (TBS) overnight at 4°C. The membranes were then incubated with secondary antibodies (1 : 2000, 7074, Cell Signaling Technology) at room temperature for 2 h. The membranes were then analyzed with enhanced chemiluminescence reagent (Biyuntian, China).

### 2.5. Real-Time Quantitative RT-PCR

After transfection for 48 h, total RNA was extracted using TRIzol™ Reagent (Lifetech, Thermo Fisher Scientific, Inc.) according to the manufacturer's protocol. Before RNA was reverse-transcribed into cDNA using RevertAid™ First Strand cDNA Synthesis Kit (Fermentas, Canada), the RNA concentration was measured by NanoDrop ND1000 (ThermoFisher, USA). The primers were designed with Beacon Designer 7.90.  Table 1 provides the primers used in this study. Target genes were obtained by qPCR using ABI StepOne, and a PCR reaction system was established as follows: 5 *µ*L of 2 × SYBR Green Master Mix, 0.25 *µ*L of forward primer, 0.25 *µ*L of reverse primer, 0.2 *µ*L of ROX, 0.5 *µ*L of cDNA template, and 3.8 *µ*L of nuclease-free water. The PCR conditions were as follows: 2 min at 50°C; followed by 40 cycles of 10 min at 95°C, 15 s at 95°C, and 30 s at 60°C; and 15 s at 95°C, 30 s at 60°C, and 15 s at 95°C. The relative quantities of NTN and Lmx1*α* mRNA were normalized to that of GAPDH mRNA.

### 2.6. Immunofluorescence

After fixing with paraformaldehyde for 30 min, the cells were blocked with 5% sheep serum for 60 min before permeation in 0.2% Triton X-100 for 20 min. Then, the cells were incubated with primary antibodies overnight at 4°C: tyrosine hydroxylase (TH; 1 : 200, ab75875, Abcam) and dopamine transporter (DAT; 1 : 400 dilution, Sc-32259, Santa). After the cells were washed four times with 0.01 mol/L of phosphate-buffered saline-Tween (PBST) for 5 min each, they were incubated at room temperature for 2 h with fluorescence-labeled secondary antibodies: anti-rabbit IgG (1 : 1000, 072-01-15-06, KPL) and anti-mouse lgG (1 : 1000 dilution, 072-03-18-06, KPL). The antibodies were rinsed again with PBST, and the slides were sealed with 50 *µ*L of 4, 6-diamidino-2-phenylindole (DAPI). The slides were photographed with an ortho-fluorescence microscope.

### 2.7. Flow Cytometry

The cells were digested by 0.25% trypsin, collected, and resuspended with 1 mL of 4% paraformaldehyde for 3 min. The cells were resuspended in PBS containing 0.1% Triton X-100 and permeabilized for 30 min. The collected cells were then sealed with 5% sheep serum at 37°C for 30 min, and the supernatant was discarded after centrifugation. The cells were resuspended with primary antibodies against sex determining region Y-box 1 (SOX1; ab109290, Abcam), paired box gene 6 (Pax6; ab5790, Abcam), and octamer-binding protein (Oct4; ab181557, Abcam) at a 1 : 500 dilution with 3% goat serum at 37°C for 1 h. The cells were then treated with secondary antibodies diluted to 1 : 1000 using 3% goat serum for 30 min, after which the fluorescence of the PBS-resuspended cells was measured by flow cytometry.

### 2.8. ELISA

To assess the function of Lmx1 and NTN overexpression, ELISA was performed to compare the dopamine levels in the control and transfected groups (Cusabio, Wuhan, China). After h-BMSCs were transfected with NTN and Lmx1*α* recombinant lentiviruses, protein was collected on day 21 and stored at −80°C for the above test.

### 2.9. Whole-Cell Patch-Clamp Recordings

Cells were rested for 30 min, perfused (2 mL/min) with extracellular fluid (140 mm NaCl, 5.4 mm KC1, 1.8 mm CaCl_2_, 1 mM MgCl, 10 mm *N*-2-hydroxyethylpiperazine-*N*-2′-ethanesulfonic acid (HEPES), and 11.1 mm glucose dissolved in 800 mL of ultrapure water with the pH adjusted to 7.4 with NaOH) to remove the surrounding impurities. The microelectrode was pushed into the cell at 45°, and the change in microelectrode resistance is detected at all times during the process. When the electrode comes into contact with the cell, the positive pressure is relieved and stabilized for 5–10 s. Once the seal resistance was higher than 1GΩ, the cell was given fast capacitance compensation, and after 2 min of stabilization, a closed connection is formed. Then, we disrupted the cell membrane and obtained whole-cell recordings.

### 2.10. Human Notch Signaling Pathway PCR Array

RT^2^ profiler™ PCR array of the human Notch signaling pathway was purchased from SA Biosciences. Total RNA was isolated from cells using TRIzol (Invitrogen). RNA samples were purified using the RNeasy™ MinElute Kit (Qiagen). SuperScript III Reverse Transcriptase (Invitrogen) was used for cDNA synthesis. The cDNAs were used for RT^2^ profiler PCR array with RT^2^ SYBR Green ROX qPCR Master Mix (Qiagen). Fold change in the mRNA expression levels between groups was calculated by 2^−ΔΔCt^.

### 2.11. Statistical Analysis

Statistical analyses were conducted using IBM SPSS Statistics (version 18) and GraphPad Prism (version 8). A two-tailed Student's *t* test was used to test for statistical differences in group means between the control and overexpression groups. *P* values of <0.05 were considered statistically significant.

## 3. Results

### 3.1. Transfection Efficiency of Lmx1*α* and NTN Recombinant Lentivirus

**Figure 1:**
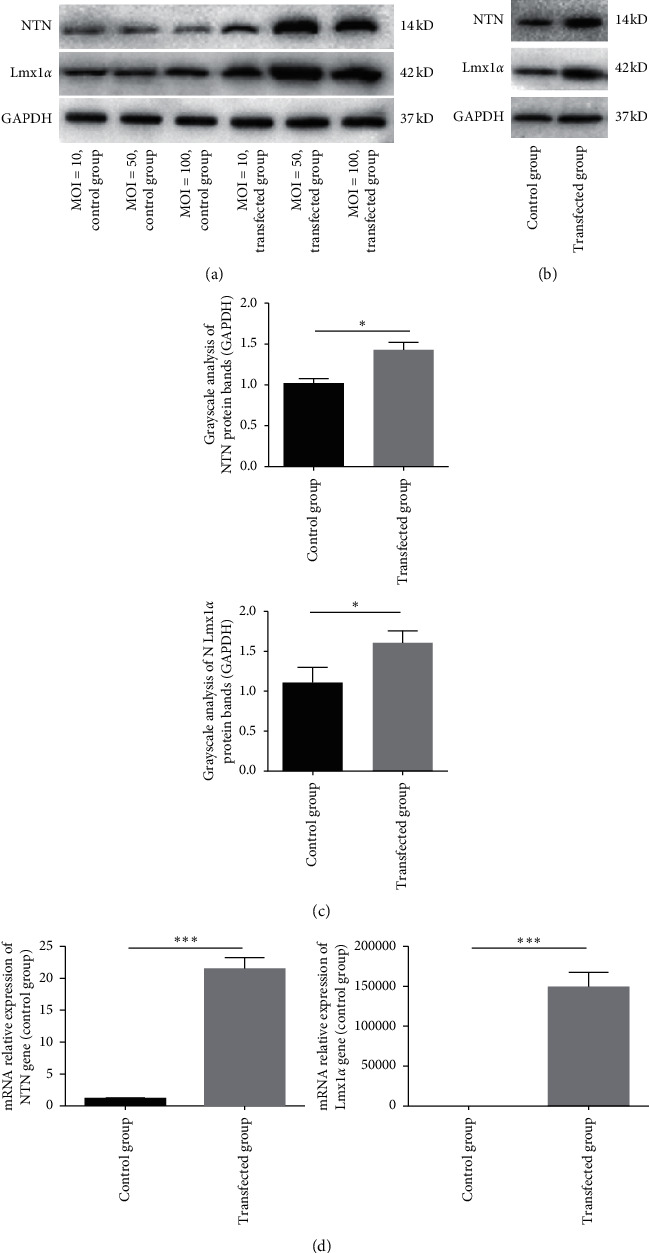
Gene transfection adopted by recombinant lentivirus to overexpress NTN and Lmx1*α*. (a) Protein expression was the most abundant when MOI was 50. (b)–(d) Western blot analysis, grayscale analysis, and PCR analysis verified significantly high expression of NTN and Lmx1*α* in the transfected group relative to the control group. GAPDH was used as an internal control. NTN, neurturin; Lmx1*α*, LIM homeobox transcription factor 1*α*; GAPDH, glyceraldehyde-3-phosphate dehydrogenase; MOI, multiplicity of infection. ^*∗*^*P* < 0.05. ^*∗∗∗*^*P* < 0.001.

When h-BMSCs were infected with NTN and Lmx1*α* recombinant lentiviruses for 48 hours, protein expression was the most abundant when MOI was 50 (Figure 1(a)). Western blot, grayscale analysis, and PCR demonstrated that the NTN and Lmx1*α* expression levels significantly differed between the transfection and control groups (*p* < 0.005; Figures 1(b)–1(d)). These results confirm the successful transfection and efficient expression of the NTN and Lmx1*α* in h-BMSCs.

### 3.2. Identification of h-BMSC-Derived Dopaminergic Neurons

**Figure 2:**
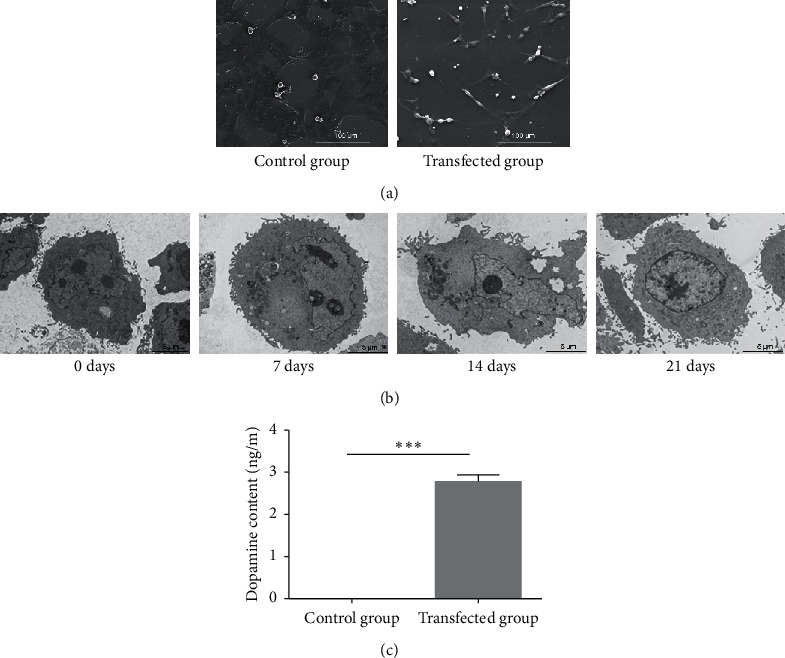
Identification of dopaminergic neurons differentiated from h-BMSCs. (a) Scanning electron microscopy showing ultrastructural characteristics of the transfection group changed after 21 days of induction. Scale bar = 100 *μ*m. (b) Transmission electron microscopy results showing ultrastructural characteristics of the transfection group changed after 7, 14, and 21 days. Scale bar = 5 *μ*m. (c) Dopamine expression of the transfection group significantly increased relative to the control group after 21 days of incubation. ^*∗∗∗*^*P* < 0.001.

The results of the scanning electron microscopy revealed that most of the neurons in the induction group appeared different compared to those in the control group. The shape of the neurons changed after 21 days of induction and could be visualized as strong stereoscopic appearance, synaptic connections, and neural network; no similar change in shape was observed in the controls (Figure 2(a)). Transmission electron microscopy showed clear nuclei, lobed nucleoids, and visible microvilli in the control group, while decreased organelles after 14 days of induction (Figure 2(b)). After 21 days of induction, inconspicuous nuclei and relatively large numbers of mitochondria were observed (Figure 2(b)). These morphological features suggested that h-BMSCs of the control group are metabolically active and exhibited poor differentiation and that neuronal differentiation was induced in the PLV-duaI-Lmx1*α*-NTN-infected h-BMSCs.

**Figure 3:**
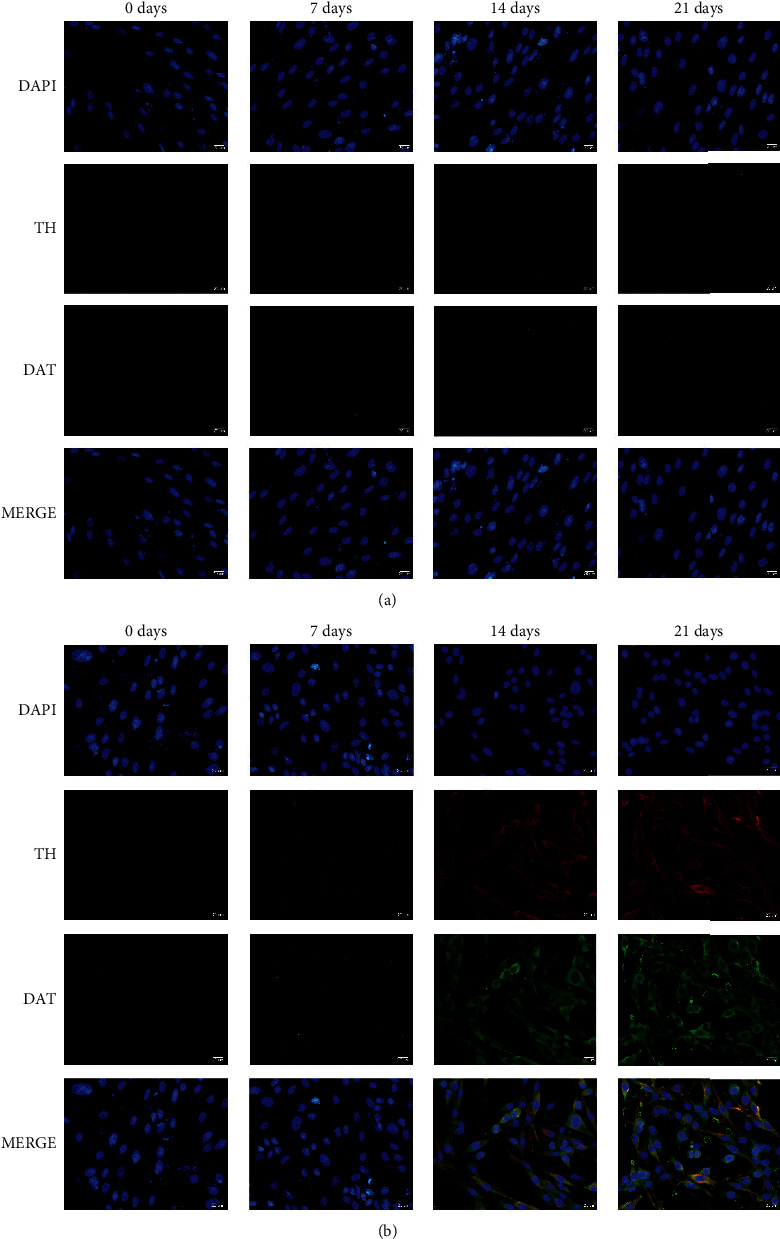
Immunofluorescence analysis showing TH (red fluorescence) and DAT (green fluorescence) expression of the transfection group was increased after 14 and 21 days of induction compared to the corresponding levels in the control group. Scale bar = 20 *μ*m. TH, tyrosine hydroxylase; DAT, dopamine transporter.

TH and DAT immunofluorescence were performed to verify whether NTN and Lmx1*α* overexpressions could promote the differentiation of dopaminergic neurons from h-BMSCs. As shown in Figure 3, TH and DAT were overexpressed in h-BMSCs after 14 and 21 days of induction, respectively, compared to the corresponding levels in the control group. Dopamine expression was significantly increased in the transfection group relative to the control group after 21 days of incubation (Figure 2(c)).

**Figure 4:**
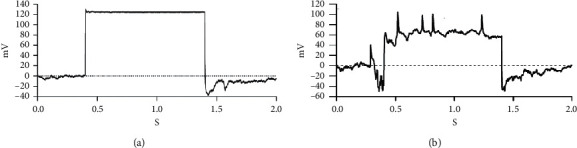
Whole cell membrane clamp recordings showing the electrophysiological behavior of the transfected group (b) presenting neuron waveform after 21 days of induction, compared to the control group (a).

The action potential was detected and recorded using a whole cell membrane clamp technique using a setting of −60 mV and an electrode impedance of 2–5 MΩ. The amplitude of the control group is almost flat throughout the frequency (Figure 4(a)). In contrast, the induced group of neurons showed an increase in amplitude, presenting a typical neuron waveform (Figure 4(b)). Together, these findings indicated the capacity of DA neurons to generate action potentials.

### 3.3. Intermediate Process of Differentiation of h-BMSCs into DA Neuron-Like Cells

**Figure 5:**
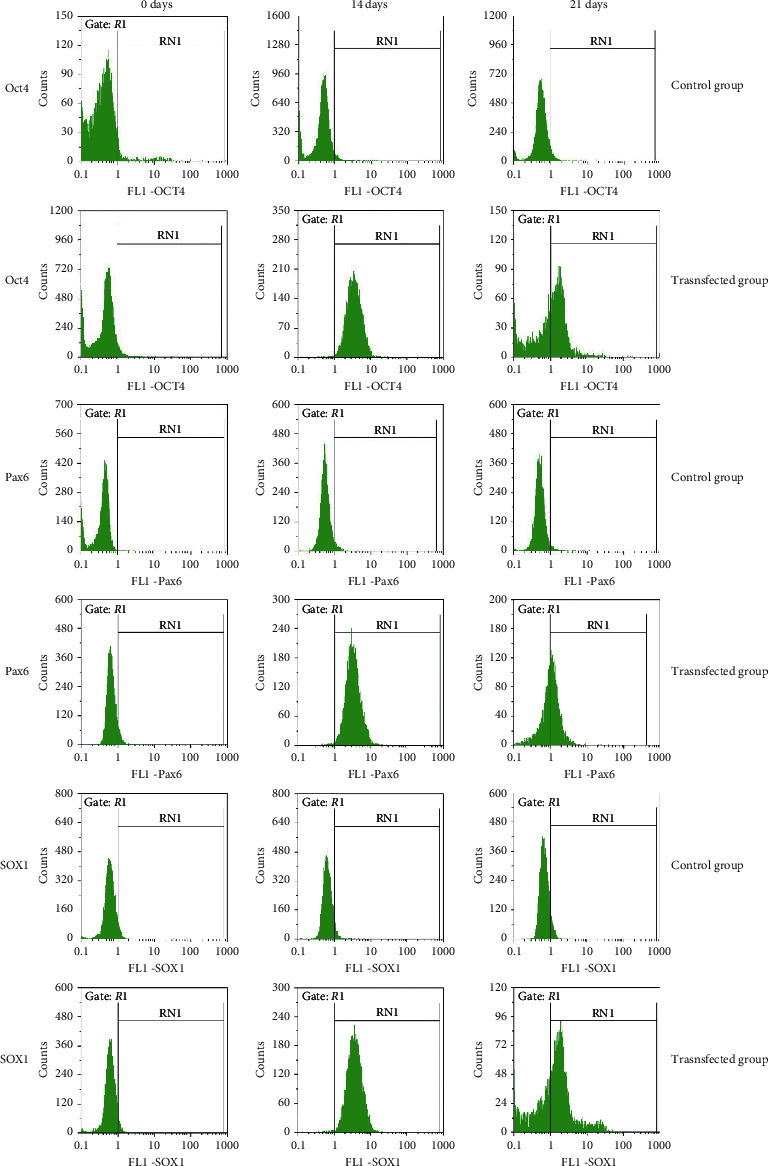
Flow cytometry revealing Oct4, Pax6, and SOX1 expressions of NSC markers increased at 14 days of incubation and gradually decreased again at 21 days of incubation. Oct4, octamer-binding protein; Pax6, paired box gene 6; SOX1, sex determining region Y-box 1; NSC, neural stem cell.

Flow cytometry revealed that Oct4 expression increased at 14 days of incubation and gradually decreased again at 21 days of incubation. The expression levels of Pax6 and SOX1 were similar to that of Oct4 (Figure 5), indicating that h-BMSCs do not directly differentiate into DA, but are subject to an intermediate process of forming the neural stem cell- (NSC-) like cells.

### 3.4. Regulation of NTN and Lmx1*α* in the Differentiation of h-BMSCs into DA

**Figure 6:**
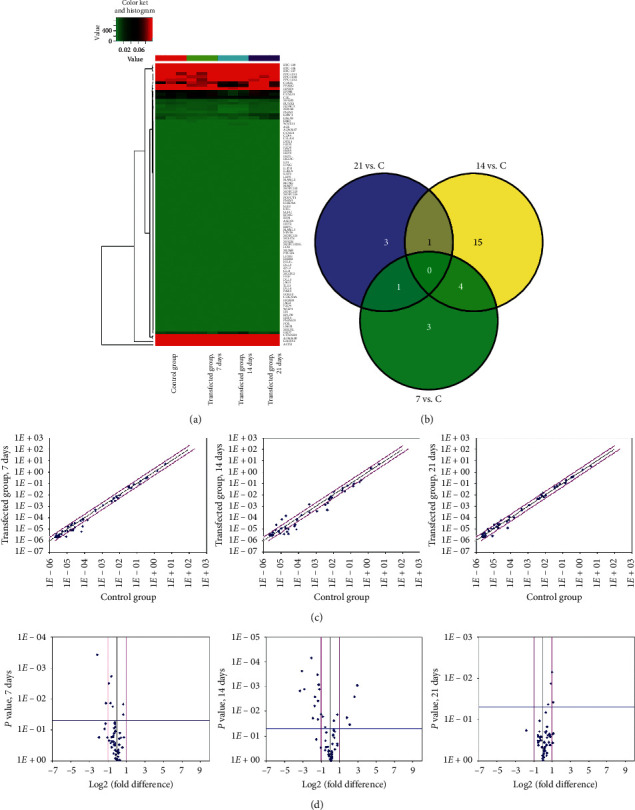
Differently expressed Notch-related genes between the control and transfected groups. (a) Heat maps showing significant changes in the expression of Notch-related genes between the control and transfected groups. (b) Venn diagram based on the differential expression of Notch-related genes between the two groups. (c) Scatter plots showing the differentially expressed Notch-related genes in the control group and transfection group. (d) Volcanic diagram showing the number of differentially expressed Notch-related genes.

We performed RT^2^ Profiler™ PCR array to further explore the mechanisms of the Notch signal pathway in the differentiation of h-BMSCs into DA. There was a significant difference in expression of related genes between the control and induction groups at 7, 14, and 21 days (Figures 6(a) and 6(b)). The correlation in Notch-related genes between the control and induction groups at 7, 14, and 21 days was examined using a scatter plot (Figure 6(c)).  Figure 6(d) and Table 2 illustrate the differential expression of genes between the two groups. These genes include genes involved in intercellular communication, cell development, and differentiation, suggesting that NTN and Lmx1*α* most likely play a role in differentiation through the indirect regulation of the Notch signaling pathway.

## 4. Discussion

PD is clinically characterized by the death of DA neurons. The treatment of PD includes drug therapy and operative management, which cannot prevent disease development. BMSCs have the ability of preventing immune rejection and secreting abundant trophic factors. BMSCs have been the focus of research to develop cell-based therapeutic strategies for PD. Recent progress in cell-based therapeutic strategies for PD is usually studied in two aspects. One is to promote the differentiation of MSCs into dopaminergic neurons and the other is to protect dopaminergic neurons. NTN is a neurotrophic factor that acts specifically on DA and motor neurons in the midbrain [5, 15, 16]. Lmx1*α* regulates all the stages of dopaminergic neuronal differentiation [7, 8, 17, 18]. Overexpression of NTN and Lmx1*α* in rhesus BMSCs has been shown to strongly promote DA neurogenesis in vitro and restore motor function after transplantation in vivo in a PD monkey model, established by treating monkeys with 1-methyl-4-phenyl-1, 2, 3, 6-tetrahydropyridin [4]. However, the mechanism by which BMSCs differentiate into DA neurons is unclear.

In present study, we chose the clinically widely used lentiviral vectors. Lentiviral vectors are characterized by stable gene expression and higher transgene payloads [19]. The focus of h-BMSCs transplantation for PD is the directed differentiation of h-BMSCs into dopaminergic neurons. The cells after induction exhibited ultrastructural characteristics of dopaminergic neuron-like cells by transmission and scanning electron microscopy. These induced cells could express TH and DAT. It is important to generate an action potential for the functional qualification of neurons. The expression of dopamine was significantly increased in the transfected group after 21 days of incubation. This means that the induced h-BMSCs are biologically able to secrete dopamine. In addition, we used patch-clamp recordings to show that the h-BMSC-derived DA neuron-like cells were capable of generating action potentials in the genetically modified h-BMSCs at 21 days of incubation, which indicates that nerve cells transmit excitement.

Ma et al. reported that NSC-like cells can be efficiently produced from h-BMSCs under appropriate culture conditions [20]. In addition, these NSC-like cells retained their capability to express molecular markers of neurons. In our study, to explore whether the differentiation of h-BMSCs to DA neurons involves neural stem cells (NSCs), we detected the expression of the NSC markers Pax6, SOX1, and Oct4. Flow cytometry analysis revealed that Pax6, SOX1, and Oct4 expression levels were elevated after 14 days in the transfection group, although these levels decreased at 21 days compared to that at 14 days. These results confirm that BMSCs differentiate into DA neurons through a process of NSC-like cells.

NTN is characterized by its capacity to nourish, protect, and repair damage to DA neurons. Lmx1*α* is a crucial factor in the development of dopaminergic neurons. However, the mechanism by which BMSCs differentiate into DA neurons is unclear. Multiple signaling pathways are involved in the differentiation of h-BMSCs into dopaminergic neurons, including SHH, Wnt, and Notch signaling pathway. No further intermediate steps involving a second messenger and protein kinase are needed during the transmission of the Notch signaling pathway. Currently, the mechanisms involved in the differentiation of BMSCs are focused on the Notch signaling pathway [21, 22]. The Notch pathway controls processes such as neurogenesis and neural stem cell maintenance [23, 24]. Therefore, the human Notch signaling pathway PCR array was performed to detect the differential expression of Notch-related genes. The Notch signaling pathway PCR array contains 84 genes, including Notch signaling pathway ligands, receptors, target genes, cell proliferation and differentiation-related genes, and neurogenesis-related genes. In addition, this array also contains other signaling pathways that intersect with the Notch signaling pathway, such as Sonic hedgehog and Wnt receptor signaling pathway member.

The five Notch ligands found in mammals are Jagged1, Jagged2, Delta1, Delta3, and Delta4 [25]. Presenilin2 (PSEN2) and ADAM metallopeptidase domain 10 (ADAM10) participate in an essential role in the proteolytic release of NICD from the Notch receptor. At 7 days of induction, the expression of Jagged1 and Presenilin2 was significantly reduced compared with the control, which showed that the Notch signaling pathway was inhibited during induction differentiation. At 14 days of induction, similar results were obtained for Jagged1, Jagged2, ADAM10, and PSEN2 expression. HES1 expression was reduced compared with that in the control group at 14 days of induction. The low expression of PSEN2 further downregulates the target genes of Notch, suggesting that multiple mechanisms are involved in the suppression of the Notch signaling pathway. These observations indicate that the expression levels of Notch signal molecules are suppressed when h-BMSCs are induced to differentiate into neural cells. However, the Notch signaling pathway is a multistep and multifactorial process.

During the differentiation of BMSCs into DA neuron-like cells, the expression of genes related to neurogenesis was changed, compared with the control. For instance, the expression of FBJ murine osteosarcoma viral oncogene homolog (FOS) was significantly increased at 7 days of induction. Fos plays an important role in neurogenesis [26]. Paired box 5 (PAX5) expression was increased at 14 days of induction, relative to the control. PAX5 acts in cell fate specification and proliferation of neural precursor cells during development [27, 28]. The expression of genes related to Wnt and SHH signaling components was also changed. The expression of frizzled family receptor 7 (FZD7) was increased and smoothened (SMO) decreased at 14 days of induction. To fully understand the role of Notch in the differentiation of h-BMSCs into neural cells, more in-depth studies are needed, especially at the molecular level.

## 5. Conclusions

Overexpression of Lmx1*α* and NTN promoted differentiation of h-BMSCs to action potential-producing DA neurons. In the process of differentiation, Lmx1*α* and NTN may affect the expression of Notch signal-related genes. In addition, h-BMSCs undergo an intermediate step of differentiating into neural stem cells before differentiating into DA neurons. Based on these findings, Lmx1*α* and NTN-expressing h-BMSCs is a promising approach for cell therapy in PD.

## Figures and Tables

**Table 1:** Primer sequences of NTN and Lmx1*α*.

Gene	Primer sequence (5′–3′)
NTN	AAAGATGGCAGGAGAGAT (forward)
	ACCAGGACATATGAATTACTAC (reverse)
Lmx1*α*	CTTGTGAACGAAGATAAGTG (forward)
	TCTACCTCTGAAGCATCC (reverse)
GAPDH	AAAGGGTCATCATCTCTG (forward)
	GCTGTTGTCATACTTCTC (reverse)

NTN, neurturin; Lmx1*α*, LIM homeobox transcription factor 1*α*; GAPDH, glyceraldehyde-3-phosphate dehydrogenase.

**Table 2:** Notch pathway qPCR results during DA neuronal differentiation.

Gene symbol	Group	Regulation	*P* value	Gene symbol	Group	Regulation	*P* value
CCND1	7 vs. C	Up	0.032	NCSTN	14 vs. C	Up	0.024
FOS	7 vs. C	Up	0.015	NEURL	14 vs. C	Down	0.044
JAG1	7 vs. C	Down	0.014	NR4A2	14 vs. C	Down	0.001
PPARG	7 vs. C	Down	0.014	PAX5	14 vs. C	Up	0.035
PSEN2	7 vs. C	Down	0.018	PPARG	14 vs. C	Down	0.004
RUNX1	7 vs. C	Down	0.002	PSEN2	14 vs. C	Down	0.001
TLE1	7 vs. C	Down	0.003	SMO	14 vs. C	Down	0.003
RPLP0	7 vs. C	Down	0.032	TLE1	14 vs. C	Down	0.001
ADAM10	14 vs. C	Down	0.001	WISP1	14 vs. C	Down	0.001
CDKN1A	14 vs. C	Down	0.019	WNT11	14 vs. C	Up	0.018
CTNNB1	14 vs. C	Down	0.013	B2M	14 vs. C	Down	0.001
EP300	14 vs. C	Down	0.001	FOSL1	21 vs. C	Up	0.013
FZD7	14 vs. C	Up	0.013	HES1	21 vs. C	Up	0.043
HDAC1	14 vs. C	Down	0.021	SNW1	21 vs. C	Up	0.007
H0XB4	14 vs. C	Up	0.003	WISP1	21 vs. C	Up	0.038
JAG1	14 vs. C	Down	0.016	RPLP0	21 vs. C	Down	0.047
JAG2	14 vs. C	Down	0.006				

C, control group; Up, upregulated; Down, downregulated.

## Data Availability

The data used to support the findings of the study are included within the article.
